# Chemical Bonding: A First-Year Seminar Series that Enhances Chemistry Majors’ Perceptions of Chemistry Subdisciplines

**DOI:** 10.1021/acsomega.5c07641

**Published:** 2025-10-26

**Authors:** Sam L. Saenger, Hannah T. Nennig, James Winters, Jacob W. Wainman

**Affiliations:** a Chemistry and Biochemistry Department, 14713 University of Minnesota Duluth, 1038 University Dr, Duluth, Minnesota 55812, United States; b Swenson College of Science and Engineering, 14713 University of Minnesota Duluth, 1303 Ordean Ct, Duluth, Minnesota 55812, United States

## Abstract

First-year seminar courses are a common strategy used to orient new college students to the skills and strategies needed to be successful in college. Here, we describe a first-year seminar series named “Chemical Bonding” that was incorporated into General Chemistry II. These seminars focused on introducing new Chemistry and Biochemistry majors to the subdisciplines of Chemistry. Faculty from 10 subfields of Chemistry led presentations of their areas of expertise, highlighting research opportunities and careers that use skills and concepts from their subdiscipline. We surveyed students to assess how their perceptions of ten subdisciplines of Chemistry were impacted by these seminars. Postseminar, students were more familiar with and understanding all subdisciplines of Chemistry. In addition, students thought the Chemistry subdisciplines were more important to their future careers, and the requirement to enroll in classes in these subdisciplines was rated as more satisfying. Some subdisciplines of Chemistry, such as Analytical Chemistry, Physical Chemistry, and Materials Science, had greater gains in perceptions. Others, including Biochemistry, Molecular Biology, and Healthcare-related sciences, showed lesser gains due to high initial perceptions of these fields. Together, these results showed that incorporating the Chemical Bonding seminar series into General Chemistry II improved students’ perceptions of the subdisciplines of Chemistry across all three years of study.

## Graphical abstract

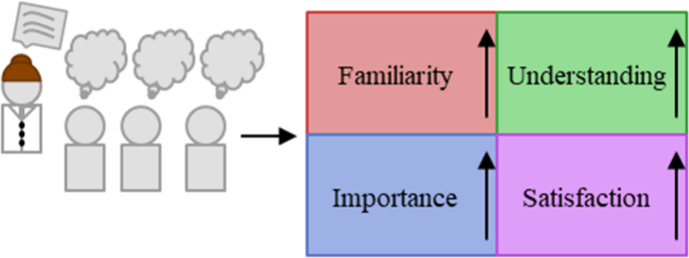

## Introduction

STEM occupations provide highly valuable contributions to society, including increased economic prosperity, national security, and rapid technological and scientific advances.^1,2^ Despite higher pay, higher rates of employment, and a greater number of available positions compared to non-STEM occupations,^1^ there is a shortage of technically trained STEM personnel in some areas of the STEM job market (particularly the public sector).^2,3^ According to the U.S. Bureau of Labor Statistics,^4^ there is a projected 8% increase in demand for chemistry and materials science positions (an estimated 7,800 projected new jobs per year) from 2023 to 2033.^4^ However, the National Science Board^5^ found that computer, mathematical, and physical sciences (including chemistry) show the lowest rates of students remaining in their chosen major from enrollment to graduation (i.e., retention) for students pursuing four-year undergraduate degrees at U.S. institutions. Of the students who pursued bachelor's degrees in these areas, only 43% graduated with degrees in these fields, while 19% moved to a different field in science and engineering.^4^ Furthermore, Rosenzweig et al.^6^ reported that 55.4% of fourth-year college students changed their majors within STEM fields at least once during their time at college, with approximately 32.5% changing their major within the first two years. Given that students who change their career plans within STEM can demonstrate lower career satisfaction and higher uncertainty in pursuing their chosen career,^6^ there is a clear and critical need to improve student retention in these majors to anticipate the future demand in the STEM job market. Thus, in 2024, the President’s Council of Advisors on Science and Technology^2^ under the Biden administration suggested that introducing first-year STEM students to STEM careers, especially those in the public sector, may increase student retention.

First-year seminars are sessions that are specifically designed to help incoming college students orient to their first year of college study. First-year seminars are well-studied tools for student retention, and their implementation has been shown to increase student satisfaction, awareness of campus resources, and general attitudes toward higher education, as well as the rate of instructor-student interactions.^7,8^ Given that there is a need for more STEM graduates, especially in the physical sciences, it seems pertinent to integrate a first-year seminar into the curriculum for STEM majors. Accordingly, there have been many types of first-year STEM seminars, which seek to achieve objectives like generally orienting students to campus life through activities and events or introducing students to their discipline to prepare them for later coursework and provide them with a network within their field.^9–13^ Other types may take a more hands-on approach with career experience, such as integrating research opportunities into the course to expose students to research early on in their academic career and to provide students the opportunity to work collaboratively with faculty and their peers.^14,15^

Despite differing objectives, these seminars typically share the same goal: increasing student retention in the university, in the major, or both. Previous studies incorporating these seminars into the STEM curriculum report results such as increased retention in STEM majors, greater confidence and alignment in scientific identity, higher first-term grade point averages, and lower rates of academic probation.^13,14^ These studies show great promise for other STEM seminars. Thiry and Weston^16^ identified that students who made an uninformed decision about their major before coming to college were less likely to persist in that major. Students who persisted in their major typically had a higher level of knowledge about their discipline when they came into college and knew more about the career opportunities in that discipline. Although, some students who persisted in their major discovered that as they learned more about their field, they realized that their goals actually did not align with their chosen field and, consequently, switched majors.^16^ Thus, these findings present an opportunity to address this career/major clarification issue in fields with lower retention rates (namely, physical sciences) using a first-year, discipline-specific seminar.

At the institution of this study, there was a noticeable decline in the retention of students in the General Chemistry sequence specific to Chemistry and Biochemistry majors. Possible causes for the lack of retention were identified anecdotally: a lack of motivation related to content (i.e., relevance)^17^ and a lack of knowledge about chemistry careers and subdisciplines. To address these struggles, a discipline-specific first-year seminar cocurriculum, dubbed “Chemical Bonding” seminars, was integrated into the two-semester General Chemistry sequence. In the first semester, the Chemical Bonding seminars focused on traditional first-year seminar content (i.e., adjusting to college life, study skills, campus resources, etc.); these seminars are not novel and are not the focus of this article.

This article presents Chemical Bonding seminars incorporated into General Chemistry II, which sought to increase students’ knowledge base (assessed as familiarity and understanding) of chemistry subdisciplines and perceived relevance of chemistry within these subdisciplines (assessed as importance and satisfaction) to students’ career goals. These seminars not only focused on the subdisciplines of chemistry defined by the American Chemical Society but also closely related interdisciplinary fields in alignment with increasing interest in interdisciplinary work across STEM.^18^

## Research Questions

The objectives of these Chemical Bonding seminars were to give students information to aid in career or major clarification and to demonstrate the relevance of an education in chemistry to their future careers. We aimed to increase students’ knowledge of the different subdisciplines of chemistry and show the respective opportunities associated with each subdiscipline. Additionally, we sought to strengthen students’ personal connection between chemistry and their future aspirations. If students perceived chemistry as being important to their personal goals, then they may also be more likely to be retained within the major. To assess the impact of the Chemical Bonding seminars, we analyzed survey data collected over a period of three years to answer the following research questions:

1. Is there an increase in students’ familiarity, understanding, sense of importance, and satisfaction for the subdisciplines of chemistry after participating in Chemical Bonding seminars?
2. Are variations observed across Chemistry subdisciplines within the Chemical Bonding seminar data?

### Chemical Bonding Seminar Design and Format

The Chemical Bonding seminar series was delivered as part of a two-semester General Chemistry lecture sequence in a section of General Chemistry reserved for Chemistry and Biochemistry majors (BA and BS), though some students (<10%) were in other related majors (e.g., Biology and Chemical Engineering) or were undeclared with interest in Chemistry and Biochemistry. At the institution of this study, General Chemistry I covers atomic and molecular structure, interactions between light and matter, stoichiometry, and thermochemistry, while General Chemistry II covers introductory physical chemistry content, including thermodynamics, kinetics, chemical equilibria, and electrochemistry (among other topics). Over the three years of this study, enrollment in these courses varied from 49 to 66 students. These classes were four credits each and met for two 110 min class periods per week for chemistry content delivery; this time was divided between active-learning-based direct instruction and small group work. There was an additional 50 min class period per week typically reserved for Chemical Bonding seminars; the laboratory component was a separate one credit course.

As mentioned previously, the Chemical Bonding seminars were implemented in both General Chemistry I and II with different objectives. The Chemical Bonding sessions in General Chemistry I focused primarily on orienting students to campus, as is typical in first-year seminar courses. As these seminars were not novel, we did not assess their effectiveness and will not describe them further in this article.

The Chemical Bonding seminars delivered in General Chemistry II were designed to improve students’ perceived familiarity, understanding, sense of importance, and satisfaction with 10 chemistry subdisciplines. We chose to have ten seminars due to availability in the course schedule and to display the breadth of chemistry and biochemistry careers. Five of the seminars focused on the subdisciplines of Chemistry as defined by the American Chemical Society: Analytical Chemistry, Biochemistry, Inorganic Chemistry, Organic Chemistry, and Physical Chemistry. Five additional subdisciplines, Chemistry Education, Environmental Chemistry, Healthcare, Materials Science, and Molecular Biology, were added to the series for three reasons: (1) we aimed to highlight interdisciplinary fields of chemistry, (2) these topics reflect the research expertise of faculty in the department at the institution where the Chemical Bonding seminars were developed, and (3) these fields capture common student interest areas.

Faculty from each subdiscipline were invited to present on the subject aligned with their expertise. Most invited presenters were colleagues within the Chemistry and Biochemistry Department; however, some faculty outside of the department were recruited as presenters for interdisciplinary topics. For example, faculty from the College of Pharmacy and Medical School copresented for the Healthcare seminar, faculty from the Biology Department introduced Molecular Biology, and faculty from Chemical Engineering led the seminar on Materials Science. These collaborations were entirely voluntary, and the 10 Chemical Bonding seminars were scheduled throughout the semester based on these faculty volunteers’ availability.

The Chemical Bonding seminars were backward designed as a collaboration between the course instructor and the faculty volunteers. This process started with developing learning objectives for each seminar. The format of these objectives was provided by the course instructor and was kept consistent across all 10 seminars for simplicity. Each blank may be filled in with a given subdiscipline.

1. Students will be able to describe what ___ is about.
2. Students will be able to identify the ___ faculty at our institution and briefly describe their research.
3. Students will be able to articulate how ___ will be useful in their future career.
4. Students will be able to list ___ courses they can expect to take for their major.

Slideshows were prepared by the 1–3 faculty volunteers in collaboration with the instructor to support the Chemical Bonding seminar presentations. During the seminar, faculty described their personal stories, sharing what sparked their interest in chemistry and their journey to their current career. They then addressed the first learning objective by displaying images that depicted generalized overviews of their subdisciplines and speaking about their chemistry subdiscipline. For example, Inorganic Chemists displayed the periodic table and then described how their field involves studying more elements than carbon. Biochemists displayed metabolic networks to demonstrate how chemistry can help us make sense of complex biological systems.

Next, the invited faculty described their research and that of colleagues from their subdisciplines, highlighting undergraduate research opportunities. The faculty also led discussions about how their subdisciplines might apply to jobs or careers. For example, Analytical Chemists emphasized the connection to forensics, and Healthcare researchers demonstrated how foundational study in Chemistry and Biochemistry served as a strong preparation for professional and graduate programs connected to human health. Finally, the seminar concluded by listing the courses in the Chemistry and Biochemistry degree plans that aligned with each subdiscipline. In some subdisciplines with no required courses (e.g., Materials Science), available electives were described (e.g., Polymer Chemistry). With any remaining class time, students were encouraged to come to the front of the class to introduce themselves to the invited speakers and ask questions.

Generally, participation in the Chemical Bonding series was received well by faculty volunteers. Most cited the fact that there was a very low barrier to entry for participation and that leading a Chemical Bonding seminar provided an avenue to recruit talented undergraduate research assistants. Additionally, faculty volunteers were eager to participate in subsequent years because the disciplinary slideshows were already prepared, requiring minimal updates year-to-year (e.g., to reflect changes to research opportunities or required courses). This decreased the faculty effort needed for participation even further. If new faculty volunteered to present their subdiscipline, they often slightly adapted the pre-existing presentations instead of building them from scratch. To further limit barriers to faculty participation each year, the order and schedule of the Chemical Bonding seminars were made flexible by the course instructor, changing to accommodate faculty availability.

## Results and Discussion

To assess the impact of the Chemical Bonding seminars, we surveyed students’ perceptions of the Chemistry subdisciplines pre- and postseminar in four dimensions listed in Table 1. Survey administration logistics are described in more detail in the Methods Section.

**Table 1:** Chemical Bonding Survey Dimensions

**Dimension**	**Survey Item**	**Likert Scale**	
Familiarity	How familiar with ___ were you before/after the Chemical Bonding seminar?	Not at all familiar (1)	Extremely familiar (5)
Understanding	To what degree did you understand what ___ was before/after the Chemical Bonding seminar?	Very poorly (1)	Excellently (5)
Importance	How important did you think __ was to your future before/after the Chemical Bonding seminar?	Not at all Important (1)	Extremely important (5)
Satisfaction	How satisfied are you with the requirement to take ___ courses as part of your major before/after the Chemical Bonding seminar?	Not at all Satisfied (1)	Extremely satisfied (5)

### Research Question 1: Do Chemical Bonding Seminars Increase Students’ Perception of Chemistry?

To address Research Question 1, we pooled student responses from all 10 seminars and plotted these data as violin plots (Figure 1). Additionally, we calculated the average pre- and postseminar survey responses for each individual subdiscipline and for the composite data; this is shown in Table 2.

**Figure 1:**
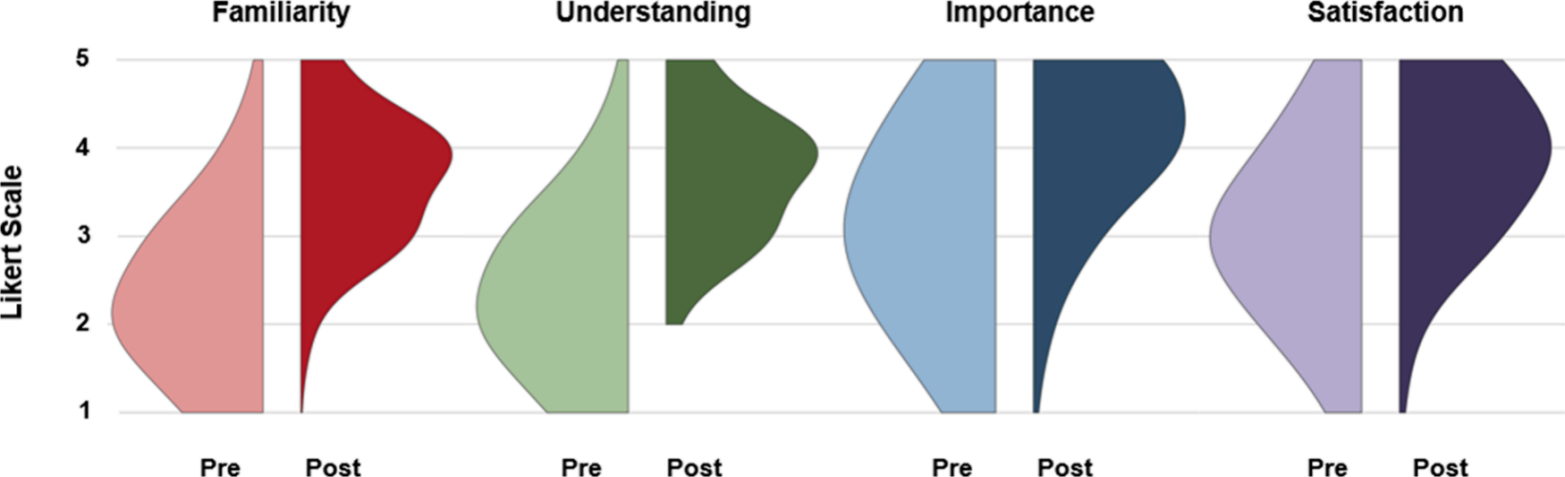
Violin Plots showing pre- and postseminar survey responses for all four dimensions, combined across all 10 subdisciplines (*n* = 1188). Red = familiarity, green = understanding, blue = importance, and purple = satisfaction. The lighter and darker shades of each color represent the pre- and postseminar responses.

**Table 2:** Survey Results^a^

		**familiarity**				**understanding**			
**subdiscipline**	***n***	**pre-**	**post-**	**%Δ**	***p***	**pre-**	**post-**	**%Δ**	***p***
Analytical	121	2.1 ± 0.2	3.7 ± 0.1	76%	5.9 × 10^–29^	2.2 ± 0.2	3.8 ± 0.1	75%	1.3 × 10^–30^
Biochemistry	126	2.9 ± 0.2	3.8 ± 0.1	32%	4.6 × 10^–16^	2.9 ± 0.1	3.9 ± 0.1	36%	6.6 × 10^–18^
Inorganic	132	2.0 ± 0.1	3.5 ± 0.1	77%	1.9 × 10^–30^	2.1 ± 0.2	3.6 ± 0.1	72%	3.7 × 10^–29^
Organic	125	2.4 ± 0.1	3.6 ± 0.1	51%	6.9 × 10^–25^	2.3 ± 0.2	3.7 ± 0.1	58%	1.8 × 10^–25^
Physical	133	2.0 ± 0.1	3.5 ± 0.1	69%	6.2 × 10^–35^	2.1 ± 0.1	3.4 ± 0.1	65%	8.1 × 10^–32^
Chem Educ	122	2.3 ± 0.2	3.9 ± 0.1	67%	9.4 × 10^–28^	2.5 ± 0.2	4.0 ± 0.1	60%	3.4 × 10^–26^
Environmental	123	2.4 ± 0.1	3.7 ± 0.1	58%	1.8 × 10^–27^	2.4 ± 0.1	3.7 ± 0.1	53%	8.9 × 10^–26^
Healthcare	42	3.3 ± 0.3	4.0 ± 0.2	24%	1.2 × 10^–04^	3.1 ± 0.3	4.0 ± 0.2	28%	6.2 × 10^–05^
Materials Sci	134	1.8 ± 0.1	3.5 ± 0.1	91%	2.8 × 10^–34^	1.9 ± 0.1	3.6 ± 0.1	90%	3.8 × 10^–36^
Mol Biology	130	2.6 ± 0.2	3.7 ± 0.2	45%	2.5 × 10^–20^	2.5 ± 0.2	3.8 ± 0.1	50%	5.2 × 10^–25^
Composite	1188	2.3 ± 0.1	3.7 ± 0.1	59%	4.7 × 10^–221^	2.4 ± 0.1	3.7 ± 0.1	59%	2.5 × 10^–224^

		**importance**				**satisfaction**			
**subdiscipline**	***n***	**pre-**	**post-**	**%Δ**	***p***	**pre-**	**post-**	**%Δ**	***p***
Analytical	121	3.0 ± 0.2	4.3 ± 0.1	41%	1.6 × 10^–16^	3.0 ± 0.2	3.9 ± 0.1	31%	7.5 × 10^–15^
Biochemistry	126	3.9 ± 0.2	4.5 ± 0.1	17%	6.8 × 10^–08^	3.7 ± 0.2	4.4 ± 0.1	16%	2.4 × 10^–06^
Inorganic	132	2.9 ± 0.2	4.1 ± 0.1	39%	2.7 × 10^–17^	3.0 ± 0.2	4.0 ± 0.1	31%	3.2 × 10^–14^
Organic	125	3.5 ± 0.2	4.3 ± 0.1	24%	5.0 × 10^–13^	3.1 ± 0.2	3.9 ± 0.1	28%	1.3 × 10^–12^
Physical	133	2.9 ± 0.2	4.2 ± 0.1	46%	3.5 × 10^–23^	2.8 ± 0.1	3.7 ± 0.1	35%	4.9 × 10^–16^
Chem Educ	122	2.9 ± 0.2	4.0 ± 0.2	36%	7.9 × 10^–12^	2.9 ± 0.2	3.7 ± 0.2	25%	5.5 × 10^–09^
Environmental	123	3.0 ± 0.2	3.8 ± 0.2	28%	2.1 × 10^–09^	3.0 ± 0.2	3.7 ± 0.2	23%	8.1 × 10^–08^
Healthcare	42	3.7 ± 0.4	4.0 ± 0.4	8%	3.2 × 10^–01^	3.6 ± 0.4	4.0 ± 0.4	12%	1.1 × 10^–01^
Materials Sci	134	2.5 ± 0.2	3.7 ± 0.2	50%	2.4 × 10^–19^	2.8 ± 0.2	3.6 ± 0.1	32%	5.2 × 10^–13^
Mol Biology	130	3.3 ± 0.2	4.1 ± 0.2	23%	4.0 × 10^–09^	3.1 ± 0.2	4.0 ± 0.2	27%	2.8 × 10^–10^
Composite	1188	3.1 ± 0.1	4.1 ± 0.1	32%	1.3 × 10^–98^	3.1 ± 0.1	3.9 ± 0.1	26%	3.4 × 10^–83^

a Pre- and postseminar means with 95% confidence intervals are provided for each subdiscipline; composite represents the pre- and postseminar means for all 10 subdisciplines combined (i.e., the data shown in Figure 1). Relative pre-to-post change (%Δ) is given as a percent increase. The provided *p*-values were determined using the Mann–Whitney U Test (see the Methods section) to compare pre- and postseminar means; *p*-values greater than 0.05 (gray) were considered insignificant.

Figure 1 and Table 2 show that in the composite data combining the survey results from all subdisciplines’ Chemical Bonding seminars, students’ perceptions of Chemistry shift to statistically significantly higher values for all dimensions surveyed. The magnitudes of these shifts were not identical across all dimensions. The dimensions of Familiarity and Understanding showed the greatest relative changes (59% and 59%, respectively), while Importance and Satisfaction showed lesser relative changes (32% and 26%, respectively). Familiarity and Understanding also shared similar averages from pre- to postseminar, with changes from 2.3 to 3.7 and 2.4 to 3.7, respectively. This result was expected because the Familiarity and Understanding dimensions are linked (i.e., being familiar with a topic is necessary to understanding it).^19,20^ Likewise, the average changes in pre- to postseminar responses for Importance and Satisfaction, from 3.1 to 4.1 and 3.1 and 3.9, respectively, are similar to each other. Again, this result was expected because students’ sense of Importance and Satisfaction are also related (i.e., believing a topic is important to you means you might be more likely to be satisfied with learning more about that topic).^17,21^ Additionally, these results revealed that the Chemical Bonding seminars had an overall greater influence on students’ knowledge base than their personal connection to chemistry.

### Research Question 2: Are Variations Observed Across Chemistry Subdisciplines within the Chemical Bonding Seminar Data?

To address Research Question 2, we created Sankey diagrams showing how students’ responses to the survey items changed from pre- to postseminar. Figure 2 shows the Sankey diagrams created for the Familiarity dimension for each chemistry subdiscipline; see Figures S1–S3 for Sankey diagrams of the other three dimensions. In these diagrams, the thickness of each curved line represents the number of students transitioning from a certain preseminar survey response to a certain postseminar survey response. Therefore, curved lines sloping upward indicate students whose familiarity increased because of the Chemical Bonding seminar. Downward slopes indicate decreased familiarity, and flat lines indicate no pre-to-post change in familiarity.

**Figure 2:**
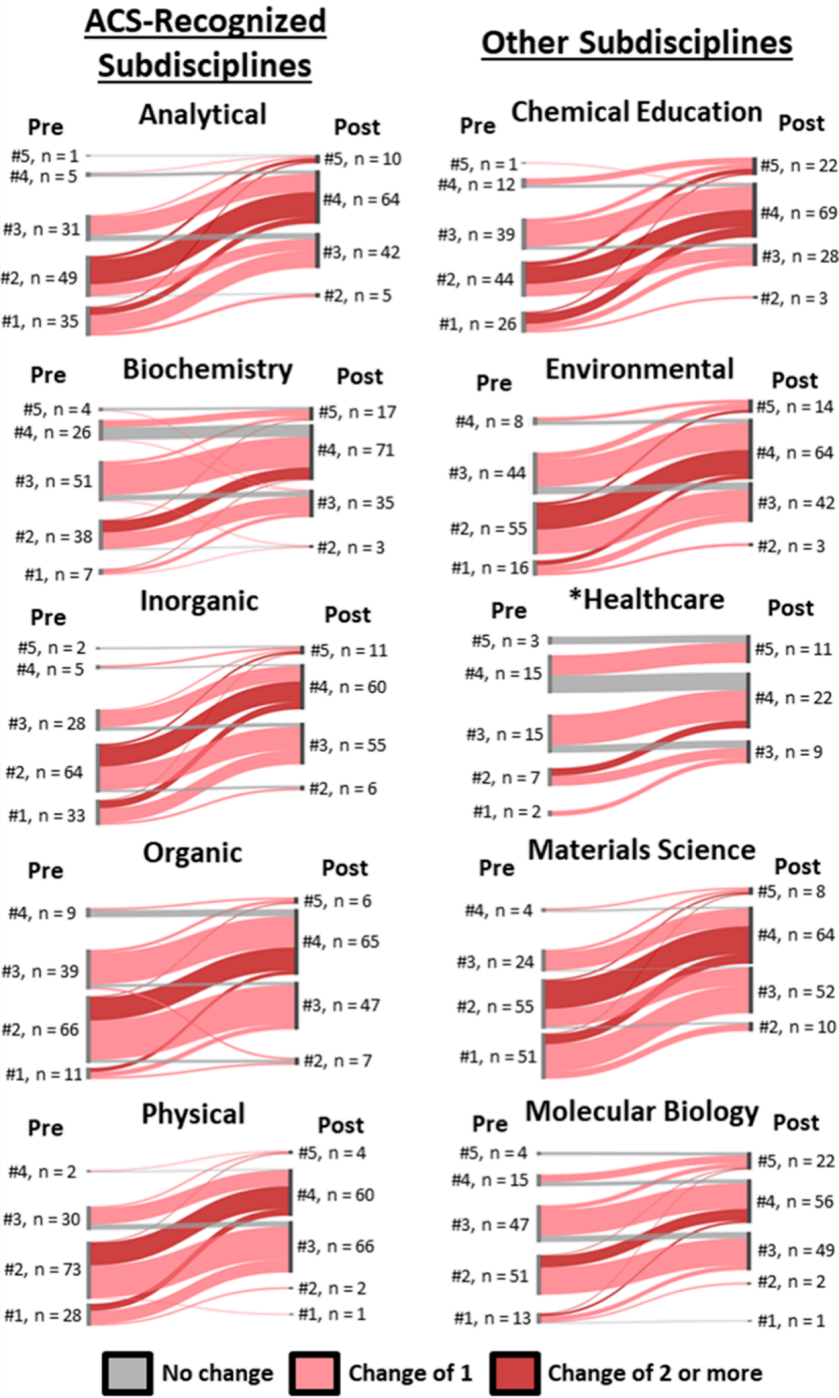
Pre-to-postseminar changes in students’ familiarity for each chemistry subdiscipline. Preseminar responses are shown to the left of each plot; postseminar responses are to the right. The number of students responding to each Likert scale is given. Gray curves indicate students who demonstrated no change in Familiarity pre to post, light red curves indicate students who changed slightly pre to post (i.e., up or down 1 Likert scale), and dark red curves indicate students who changed more pre to post (i.e., up or down 2 or more Likert scales). The asterisk on the Healthcare subdiscipline denotes that it was added in only the last year of the study, explaining the lower *n* values.

Figure 2 showed that students’ survey responses generally trended upward pre-to-post, indicating that the Chemical Bonding seminars increased their Familiarity across each subdiscipline. Likewise, pre-to-post increases dominated the Sankey diagrams for the other surveyed dimensions of Understanding, Importance, and Satisfaction (see Figures S1–S3). These results mirror the subdiscipline-specific means shown in Table 2.

There were noticeable differences in the Familiarity results when comparing across the Chemistry subdisciplines. By considering the students involved in the surveys, we speculated about the origins of the observed variations. Students reported more familiarity with Biochemistry, Healthcare, and Molecular Biology (with preseminar averages of 2.9, 3.3, and 2.6, respectively) prior to the Chemical Bonding seminars. This result was likely due to many students’ career goals overlapping with those of these fields (i.e., professional or graduate school). Additionally, 63% of students in this course were Biochemistry majors (BA or BS), which notably required coursework in biochemistry and biology, explaining why our results show increased initial familiarity with these subdisciplines. Students were less familiar with the other subdisciplines before the seminar. Analytical Chemistry, Physical Chemistry, and Materials Science were the least familiar to students initially (with preseminar averages of 2.1, 2.0, and 1.8, respectively). This result is likely due to a lack of exposure to and/or coursework in those subdisciplines. Two of these subdisciplines, Analytical Chemistry and Materials Science, also had the largest changes in Familiarity compared to the other subdisciplines, with the relative changes of 76 and 91% respectively.

Trends similar to those observed in the Familiarity dimension were also observed in the Understanding dimension (see Figure 2, Figure S1, and Table 2). Students rated their initial understanding of Biochemistry, Chemical Education, Healthcare, and Molecular Biology (preseminar averages of 2.9, 2.5, 3.1, and 2.5, respectively) higher than other chemistry subdisciplines. Their initial understanding of Inorganic Chemistry, Physical Chemistry, and Materials Science was rated lower (preseminar averages of 2.1, 2.1, and 1.9, respectively). Students’ overall changes in understanding were the greatest for Analytical Chemistry, Inorganic Chemistry, and Materials Science, with the relative changes being 75, 72, and 90%, respectively. The fact that the familiarity and understanding results align well was expected; if students are initially familiar with a subdiscipline, it can be expected that they also understand more about that subject and vice versa.^19,20^

For the dimensions of Importance and Satisfaction (see Figures S2 and S3 and Table 2), there were noticeably more students whose pre- to post-responses exhibited no change or only small changes (i.e., 1 Likert scale) when compared to the Familiarity and Understanding items. The subdisciplines that showed the largest percent changes in Importance were Analytical Chemistry (41%), Physical Chemistry (46%), and Materials Science (50%). These findings align closely with the results from Familiarity and Understanding, particularly with Analytical Chemistry and Materials Science. Analytical Chemistry, Inorganic Chemistry, Physical Chemistry, and Materials Science showed the largest percent changes in Satisfaction, with percent changes of 31, 31, 35, and 32%, respectively. Though the changes in Satisfaction from pre to post were not very large (e.g., from 3.0 to 3.9 for Analytical Chemistry), these changes were still observed for most subdisciplines, demonstrating that students’ attitudes toward these disciplines generally increase positively.

Certain subdisciplines were initially recognized as more important and satisfying than other subdisciplines, leading to smaller relative changes in the survey responses. This occurred for Biochemistry (satisfaction pre mean of 3.7, 16% change), Healthcare (satisfaction pre mean of 3.6, 12% change), and Molecular Biology (satisfaction pre mean of 3.1, 27% change). These results indicate that students generally acknowledged these subdisciplines as personally important and satisfying before the Chemical Bonding seminars and maintained these attitudes after the seminars. These results also aligned with the results from Familiarity and Understanding. Students typically familiar with and having an understanding of these subdisciplines held these subdisciplines in higher regard. Additionally, given that many students in this course aspire to pursue careers in biochemistry, healthcare, or a related field, it is reasonable that the four dimensions have higher initial scores and lower rates of overall change for these subdisciplines.

Taken together, these results suggest that the Chemical Bonding seminars influence students’ Familiarity and Understanding for each subdiscipline of chemistry to a greater extent and Importance and Satisfaction to a lesser extent. The survey items related to Familiarity and Understanding examine students’ change in knowledge on the subdiscipline, while questions related to Importance and Satisfaction examine students’ change in attitude and/or motivation toward the subdiscipline. Accordingly, these results indicate that students may learn more about a subdiscipline by participating in seminars such as the Chemical Bonding seminars we designed. However, this increase in knowledge about the subdisciplines may not necessarily always correlate with whether students enjoy that subdiscipline more or perceive it as more relevant to their future aspirations. That said, for the composite of all subdisciplines, the Chemical Bonding seminars were improved across all surveyed dimensions.

### Comparing Chemical Bonding Seminars to Other First-Year Seminars

Other first-year seminars have found success in improving factors that relate closely to students’ familiarity, understanding, importance, and satisfaction. Al-Sheeb et al.^7^ conducted a study on a non-STEM first-year seminar and found an increase in student satisfaction, student attitudes toward higher education, and awareness of campus resources, which aligns similarly with our findings related to student satisfaction, understanding, and familiarity. Myers et al.^10^ found that first-year STEM students in a career-specific, Chemical Engineering-focused seminar reported increased comfort interacting with faculty and upperclassmen, increased knowledge about the field, and an increased capability to identify their interests in their field. Delaware et al.^11^ described similar findings with a career-specific seminar for chemistry students, who demonstrated increased familiarity with faculty and their field. Our study mirrors these results, showing that first-year seminars are a strong method for improving student perceptions of a discipline. Novel to our approach, however, was the incorporation of this seminar series into a required General Chemistry course, which was an intentional design choice. By incorporating the Chemical Bonding series into General Chemistry II and assigning credit for participating in these seminars, the instructor emphasized the value of gaining this knowledge for Chemistry and Biochemistry majors.

Important to the design philosophy of the Chemical Bonding seminars was the involvement of faculty volunteers. This approach was an intentional choice to better welcome new Chemistry and Biochemistry majors into the department, giving students a chance to meet more faculty and better integrate into the departmental community. Such an approach has been studied in the context of other first-year seminars. Solano et al.^12^ found that hosting outside speakers demonstrated a significant effect on students’ understanding of the careers in their field and their ability to access people with differing careers in chemistry. These factors align with our surveyed dimensions of familiarity and understanding, which relate to expanding students’ knowledge bases in the field of chemistry. In Jablon-Roberts and McCracken’s^22^ qualitative study, they noted that students often shared the opinion that they “hold the same general expectations of guest speakers as [their] professors,” which suggests that coordination among instructors and faculty volunteers is crucial to the success of a seminar. Klatt and Ray,^13^ Tucci et al.,^9^ and Ortiz and Sriraman^23^ also studied the impact of faculty involvement and motivation on first-year seminar success. Among their findings was the conclusion that faculty identity, as well as the order and number of faculty member(s) speaking during the seminar, showed no significant impact.^9,13,23^ These results are encouraging as flexible scheduling is crucial to incorporate faculty volunteers into programming such as the Chemical Bonding seminars.

### Limitations and Implications

Our data suggest that the Chemical Bonding seminar series was successful in orienting General Chemistry students to subdisciplines in chemistry; however, the results are limited by the use of Likert-style survey items. Without a qualitative understanding of students’ experiences, we do not know how or why students’ familiarity, understanding, sense of importance, and satisfaction increased. In addition, by simply averaging the survey items, we lose nuances for subpopulations of students in the data set, particularly for groups underrepresented in the student population. For example, at the institution where this study is conducted, 85% of the General Chemistry students are white. This factor (and others) limits our interpretation of the impact of the Chemical Bonding seminars, as there may be differential impacts for students of different backgrounds. This may be a point of interest for future studies to investigate. Additionally, our study does not track survey respondents after taking the General Chemistry sequence, so we do not know whether the seminar series improved retention long-term. The exploration of these aspects of the seminar experience may allow for greater clarity on this seminar’s impact.

The interpretation and impact of our results are also limited because only one survey item was used to measure each dimension in the survey. We recognize that accurate multidimensional analysis cannot be achieved without gathering more data, including developing additional validated and reliable items to measure each dimension. Thus, we emphasize that our results, and their respective interpretations, require further investigation into how the implementation of this seminar impacts first-year chemistry students’ familiarity, understanding, sense of importance, and satisfaction.

## Conclusions

The objective of this study was to improve first-year chemistry students’ familiarity, understanding, sense of importance, and satisfaction with each subdiscipline of Chemistry by incorporating a Chemical Bonding seminar into General Chemistry II. Based on our results, it is evident that students in this seminar demonstrated an overall increase in all four dimensions across all subdisciplines. Additionally, there are interesting differences observed among the various subdisciplines and dimensions that were interpreted using additional information about the specific students who participated.

## Methods

### Survey Design and Administration

To assess the impact of these Chemical Bonding seminars on students’ perceptions of the subdisciplines, we administered surveys following each seminar presentation. These surveys were delivered to students as part of an online assignment through the learning management system (Canvas) before each Chemical Bonding seminar’s class period began, and they remained open for 1 week after each seminar. Many students completed the survey during the presentation. The 10 assignments containing the surveys were collectively worth approximately 5% of the overall score in the course, and they were graded on completion. Seminar attendance was recorded by the instructor and was required to earn a credit.

Each Chemical Bonding seminar assignment had the same format and was distributed in two parts (an example of a full assignment can be found in the Supporting Information). The first part of the assignment contained general questions about the subdiscipline of Chemistry that aligned with the four learning objectives of the seminars. (e.g., ″Briefly describe Analytical Chemistry” or “Name a faculty member who does Analytical Chemistry research”). The second part of the assignment included the eight survey items. These items asked students to report on four dimensions (familiarity, understanding, importance, and satisfaction) related to their perceptions of the chemistry subdisciplines before and after attending the Chemical Bonding seminars. These perceptions were scored using five-point Likert scales (see below, imagining the blanks are filled in with each Chemistry subdiscipline).

1. Familiarity: How familiar with ___ were you before the Chemical Bonding Activity?
  a. Not at all familiar.
  b. Slightly familiar.
  c. Moderately familiar.
  d. Very familiar.
  e. Extremely familiar.
2 Understanding: To what degree did you understand what ___ was before the Chemical Bonding Activity?
  a. Very poorly.
  b. Poorly.
  c. Fairly well.
  d. Well.
  e. Excellently.
3 Importance: How important did you think ___ was to your future before the Chemical Bonding Activity?
  a. Not at all important.
  b. Slightly important.
  c. Moderately important.
  d. Very important.
  e. Extremely important.
4 Satisfaction: How satisfied were you with the requirement to take ___ as part of being a Chemistry or Biochemistry major before the Chemical Bonding Activity?
  a. Not at all satisfied.
  b. Slightly satisfied.
  c. Moderately satisfied.
  d. Very satisfied.
  e. Extremely satisfied.

### Rationale for the Four Survey Dimensions

These four dimensions in these survey items relate strongly to the learning objectives and purpose of this study and, thus, need to be operationally defined. Familiarity is defined by Turner^24^ as having a “thorough knowledge of, or intimacy with something or someone.” Therefore, increasing the knowledge of a subject is expected to increase familiarity with that subject. Heidegger^19^ and Dreyfus^20^ establish that familiarity is a precursor to understanding, which can be defined as “know-how,” or the actual application of knowledge gained through becoming familiar with something. By increasing familiarity with a subject, it is expected that understanding also increases. Thus, we use the students’ responses to familiarity and understanding survey items as evidence of their growth in knowledge about a particular subject and their ability to apply that learned information.

Similarly, the constructs of personal importance and satisfaction also relate to each other; however, their relationships are more complex. The consensus, according to Mobley and Locke,^21^ is that satisfaction is an emotional response to an experience that is dependent on how important and valuable that experience is perceived to be by the person experiencing it. The spectrum of how satisfying an experience is can be defined as “the range of affect”. The range of affect may be larger when the experience is more important, and subsequently, the more that the experience matters, the greater the influence it can have on your satisfaction. For example, a job interview for a dream job might be considered highly important, and thus, if it goes well, it might be considered a highly satisfying experience; on the other hand, if the position is very important and the interview goes poorly, the experience might be considered highly unsatisfying. Oppositely, a job interview for a position that is of low importance to you might not greatly impact your satisfaction, whether the interview goes well, because you were not initially emotionally invested.^24^ Perceived importance and subsequently satisfaction are also linked to relevance. Frymier and Shulman^17^ note that the communication of relevance “contributes to the linkage between content and a student’s interests and goals.” That is, as content becomes more relevant to students, it is likely that it will also become more important to them.^17^ Consequently, as perceived importance increases, it can be expected that the range of effect increases, as well. In short, importance and satisfaction appear to be dependent on each other, and they seem to be closely linked to a person’s emotional investment in an experience and that experience’s perceived relevance to their future aspirations. By operationally defining these dimensions, we aim to more effectively interpret and communicate our results.

### Data Collection, Processing, and Analysis

Pre- and postsurvey responses regarding the seminar were collected for three semesters (Spring 2022, Spring 2023, and Spring 2024) of General Chemistry II. The survey data were anonymized and cleaned, focusing only on collecting responses that provided integer values (1–5) for the Likert responses. We chose the following inclusion criteria for survey responses to be included in further analysis: (1) all survey questions needed to be completed per seminar session, and (2) responses that included qualitative elements (e.g., comments) were not included for the survey analysis. Though students had the opportunity to respond after every seminar session, their responses did not always meet the criteria necessary for inclusion in the data set. Students whose responses were excluded in one subdiscipline may not be included in other subdisciplines if the inclusion criteria were satisfied in that subject and/or if they were not in attendance for the other subdiscipline’s seminar. Thus, the total number of survey responses across the subdiscipline sessions is variable.

To analyze the data, we calculated the mean for the pre- and postseminar survey responses, and we analyzed them for statistical significance (*p* < 0.050) using the Mann–Whitney U test (MWUT) in the scipy.stats Python package. Pre- and postseminar response means were first computed for a given dimension, subdiscipline, and year. We visualized the data across all dimensions, subdisciplines, and years using violin plots, which were created using the seaborn Python package. An illustrative example of this analysis is shown in Figure 3, which includes the violin plots of students’ responses for the Familiarity survey item before and after the Analytical Chemistry seminar for all three years. We found that the year-to-year preseminar responses were not distinguishable from each other (*p* > 0.050); this was also observed for year-to-year postseminar responses. Additionally, the preseminar responses were significantly different (*p* < 0.050) from the postseminar responses, and always shifted upward.

**Figure 3:**
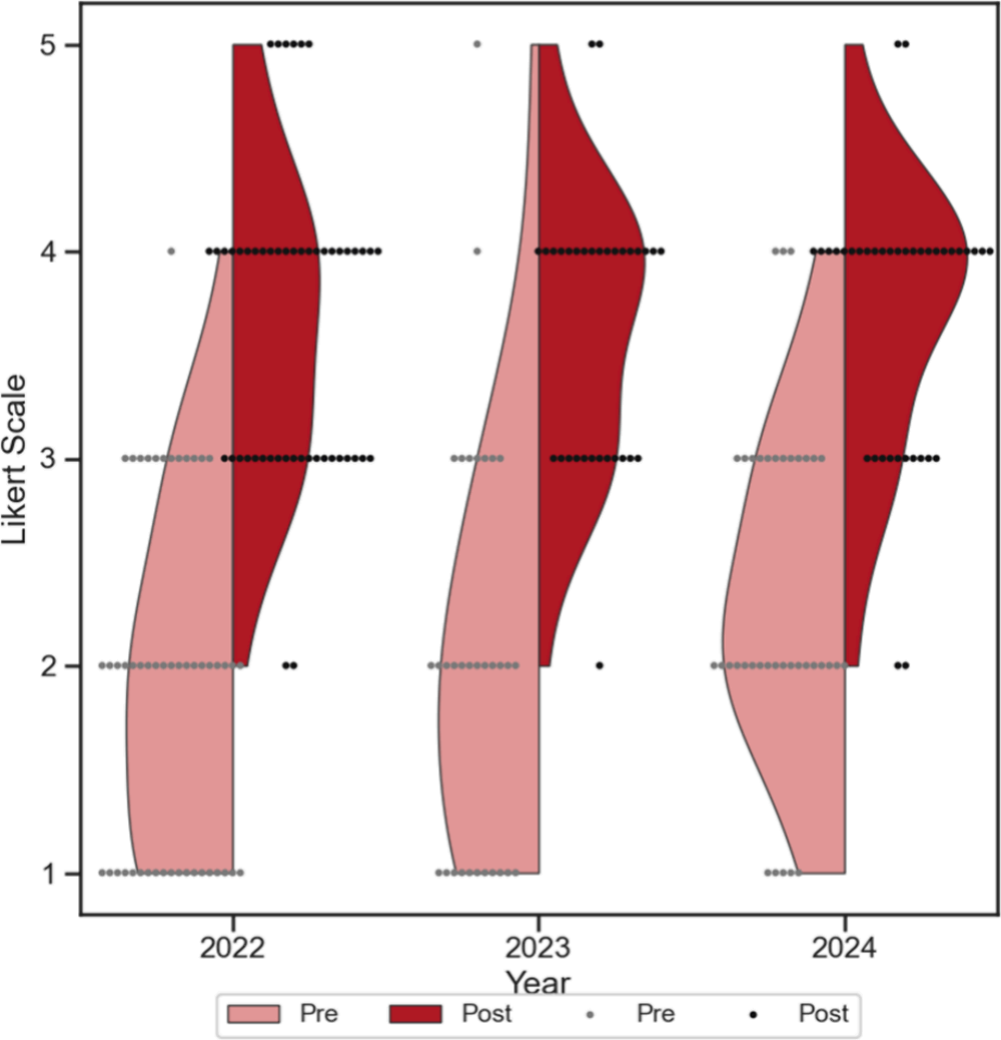
Violin plots showing year-to-year survey responses for familiarity with Analytical Chemistry. These plots demonstrate that the pre- and postseminar responses do not change between years. Light red represents the preseminar responses, while dark red represents the postseminar responses.

Taken together, these results indicate that students’ familiarity with Analytical Chemistry is similar year-to-year before participating in the Chemical Bonding series, and the seminar successfully improved their familiarity to approximately the same extent each year. These results were observed across all years, subdisciplines, and dimensions (Figures S4–S7). Given the similarity in the results, we decided to pool data from the three years together to better assess the general impacts of the Chemical Bonding series across all dimensions and subdisciplines. Importantly, the Healthcare Chemical Bonding seminar was introduced in Spring 2024; thus, the data in this article for this subdiscipline represent only one year of survey data collection.

Pre- and postseminar responses pooled over the three years of data collection were averaged for each dimension and subdiscipline. Pre- and postseminar averages were compared using the MWUT, and *p*-values from these tests are shown in Table 2. A Composite data set was created by combining the data from all chemistry subdisciplines for each survey dimension; these data were analyzed in the same fashion as the data for each subdiscipline. To calculate the relative percent change from preseminar to postseminar, we used the following equation:

$$\mathrm{relative}\;\mathrm{change}\;(\%)=\frac{{\mathrm{post}}_{\mathrm{mean}}-{\mathrm{pre}}_{\mathrm{mean}}}{{\mathrm{pre}}_{\mathrm{mean}}}\times 100\%$$

where pre_mean_ and post_mean_ represent the pre- and postseminar average values, respectively.

## Supplementary Material



## Notes

Example survey assignment and supplemental figures (PDF)
