# Supplementary material for: Chemical Bonding: A First-Year Seminar Series that Enhances Chemistry Majors’ Perceptions of Chemistry Subdisciplines
Source: ACS Omega. 2025 Oct 26;10(43):51700–8. doi: 10.1021/acsomega.5c07641 (PMC12593962; doi:10.1021/acsomega.5c07641)
Supplement: Supplementary file 1 [file ao5c07641_si_001.pdf]

Chemical Bonding: A First-Year Seminar Series that Enhances Chemistry Majors' Perceptions of Chemistry Subdisciplines

Sam L. Saenger[a], Hannah T. Nennig[b], James Winters[a], and Jacob W. Wainman[a]*

[a] Chemistry and Biochemistry Department, University of Minnesota Duluth, 1038 University Dr, Duluth, Minnesota 55812, United States

[b] Swenson College of Science and Engineering, University of Minnesota Duluth, 1303 Ordean Ct, Duluth, Minnesota 55812, United States

CHEMICAL BONDING SEMINAR ASSIGNMENT EXAMPLE

The following example assignment was made available to students through the online learning management system (Canvas). This assignment was set up to accept student responses as an open ended text box, wherein students replied to all six questions about whatever Chemical Bonding seminar they were participating in. The given example is for Analytical Chemistry, but this was substituted for the corresponding subdiscipline of chemistry.

Answer the following about Analytical Chemistry:

1) What does it mean to study this field of chemistry?
2) Name at least one professor at [*our institution*] whose research is in this area of chemistry. Describe their research in a sentence or two.
3) List one or two careers that use this type of chemistry.
4) What course(s) will you take to learn this type of chemistry?
5) Rank your perception of this field of Chemistry **BEFORE** this class period:

   Familiarity: How familiar with Analytical Chemistry were you before the Chemical Bonding seminar?
   1. Not at all familiar.
   2. Slightly familiar.
   3. Moderately familiar.
   4. Very familiar.
   5. Extremely familiar.

   Understanding: To what degree did you understand what Analytical Chemistry was before the Chemical Bonding seminar?
   1. Very poorly.
   2. Poorly.
   3. Fairly well.
   4. Well.
   5. Excellently.

   Importance: How important did you think Analytical Chemistry was to your future before the Chemical Bonding seminar?
   1. Not at all important.
   2. Slightly important.
   3. Moderately important.
   4. Very important.
   5. Extremely important.

   Satisfaction: How satisfied were you with the requirement to take Analytical Chemistry as part of being a Chemistry or Biochemistry major before the Chemical Bonding seminar?
   1. Not at all satisfied.
   2. Slightly satisfied.

3. Moderately satisfied.
4. Very satisfied.
5. Extremely satisfied.

6) Rank your perception of this field of Chemistry **AFTER** this class period:

Familiarity: How familiar with Analytical Chemistry were you after the Chemical Bonding seminar?

1. Not at all familiar.
2. Slightly familiar.
3. Moderately familiar.
4. Very familiar.
5. Extremely familiar.

Understanding: To what degree did you understand what Analytical Chemistry was after the Chemical Bonding seminar?

1. Very poorly.
2. Poorly.
3. Fairly well.
4. Well.
5. Excellently.

Importance: How important did you think Analytical Chemistry was to your future after the Chemical Bonding seminar?

1. Not at all important.
2. Slightly important.
3. Moderately important.
4. Very important.
5. Extremely important.

Satisfaction: How satisfied were you with the requirement to take Analytical Chemistry as part of being a Chemistry or Biochemistry major after the Chemical Bonding seminar?

1. Not at all satisfied.
2. Slightly satisfied.
3. Moderately satisfied.
4. Very satisfied.
5. Extremely satisfied.

SUPPLEMENTAL FIGURES

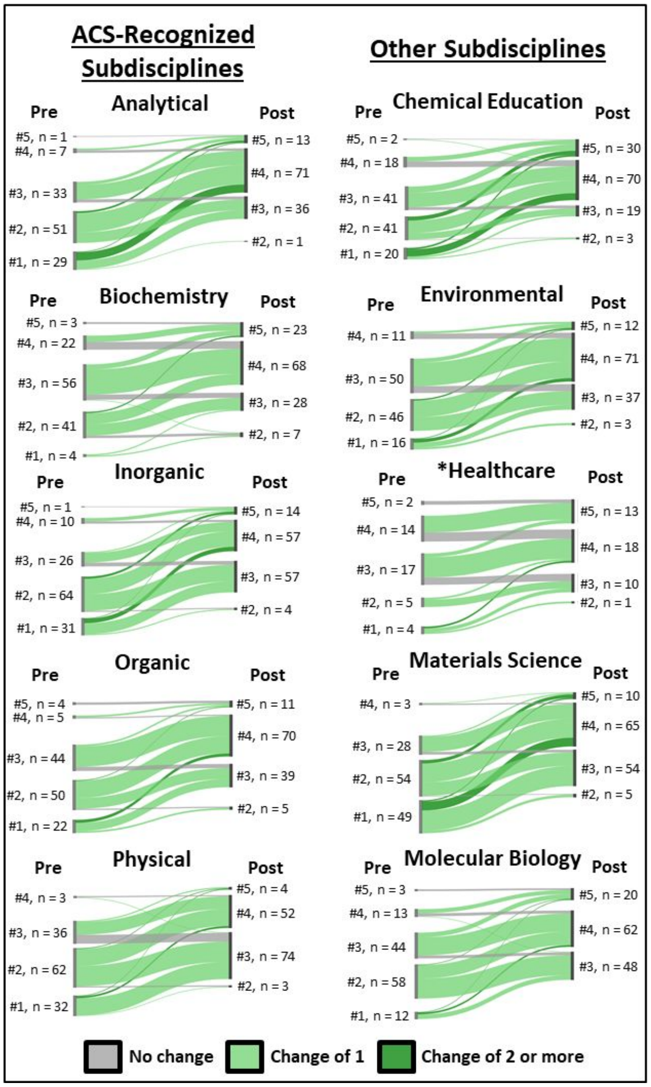

Figure S1: Pre-to-post seminar changes in students' understanding of each chemistry subdiscipline. Pre-seminar responses are shown to the left of each plot, post-seminar responses are to the right. The number of students responding with each Likert scale is given. Gray curves indicate students who demonstrated no change in Familiarity pre to post, light green curves indicate students who changed slightly pre to post (i.e. up or down 1 Likert scale), and dark green curves indicate students who changed more pre to post (i.e. up or down 2 or more Likert scales). The asterisk on the Healthcare subdiscipline denotes that it was added in only the last year of the study, explaining the lower n values.

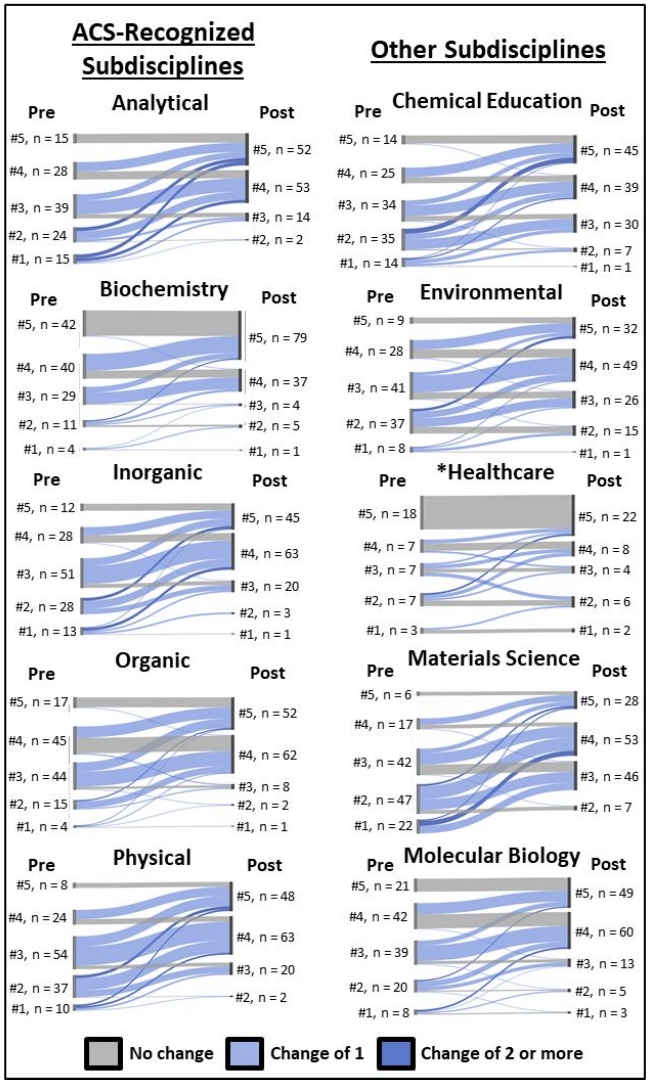

Figure S2: Pre-to-post seminar changes in students' sense of importance for each chemistry subdiscipline. Pre-seminar responses are shown to the left of each plot, post-seminar responses are to the right. The number of students responding with each Likert scale is given. Gray curves indicate students who demonstrated no change in Familiarity pre to post, light blue curves indicate students who changed slightly pre to post (i.e. up or down 1 Likert scale), and dark blue curves indicate students who changed more pre to post (i.e. up or down 2 or more Likert scales). The asterisk on the Healthcare subdiscipline denotes that it was added in only the last year of the study, explaining the lower n values.

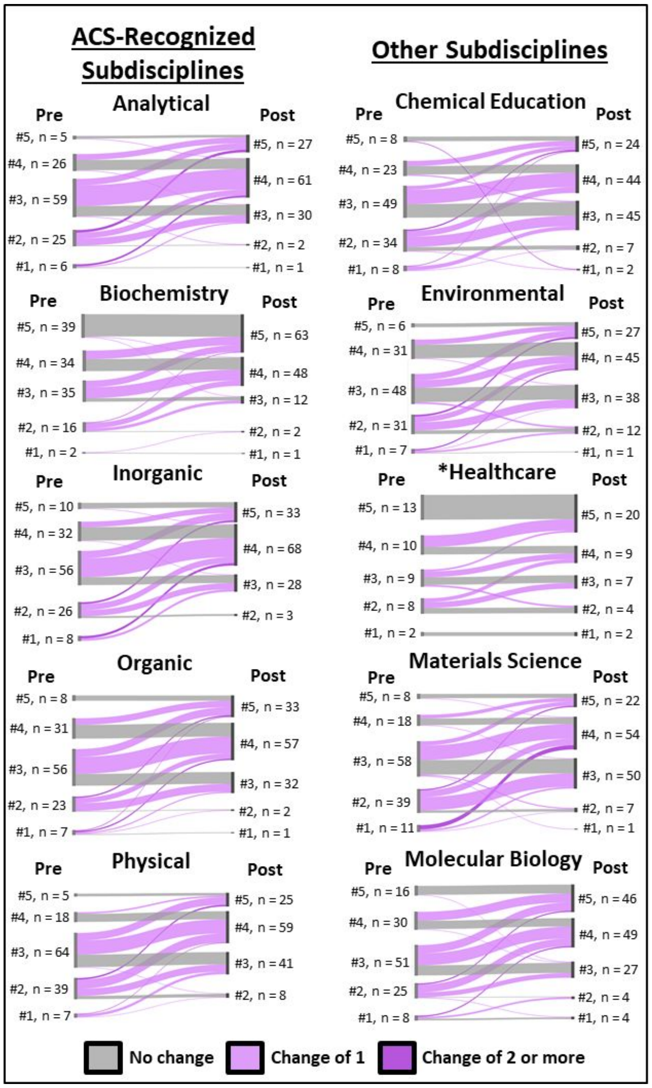

Figure S3: Pre-to-post seminar changes in students' satisfaction with each chemistry subdiscipline. Pre-seminar responses are shown to the left of each plot, post-seminar responses are to the right. The number of students responding with each Likert scale is given. Gray curves indicate students who demonstrated no change in Familiarity pre to post, light purple curves indicate students who changed slightly pre to post (i.e. up or down 1 Likert scale), and dark purple curves indicate students who changed more pre to post (i.e. up or down 2 or more Likert scales). The asterisk on the Healthcare subdiscipline denotes that it was added in only the last year of the study, explaining the lower n values.

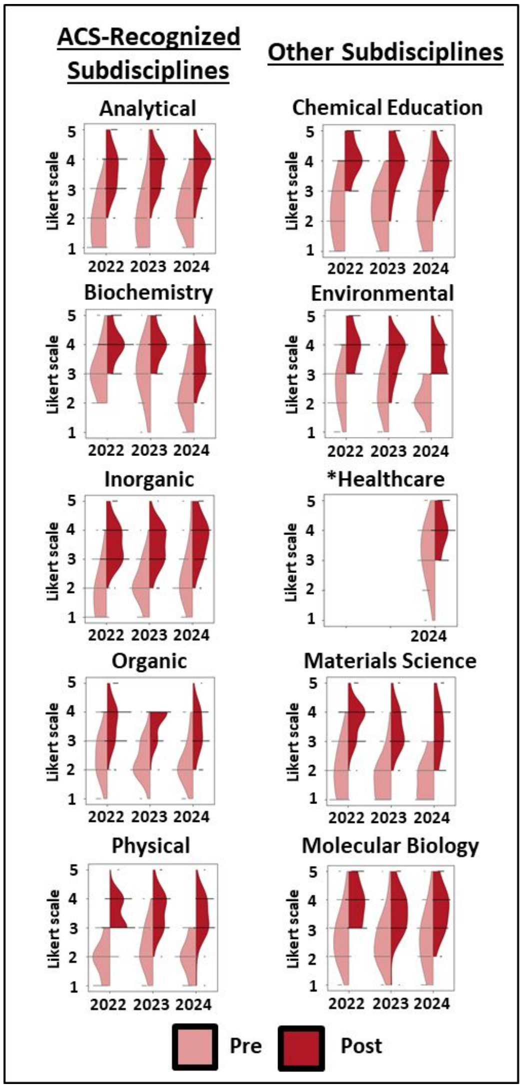

Figure S4: Year-to-year comparisons of survey responses to the Familiarity survey items. Pre-seminar responses are shown in light red, post-seminar responses are shown in dark red.

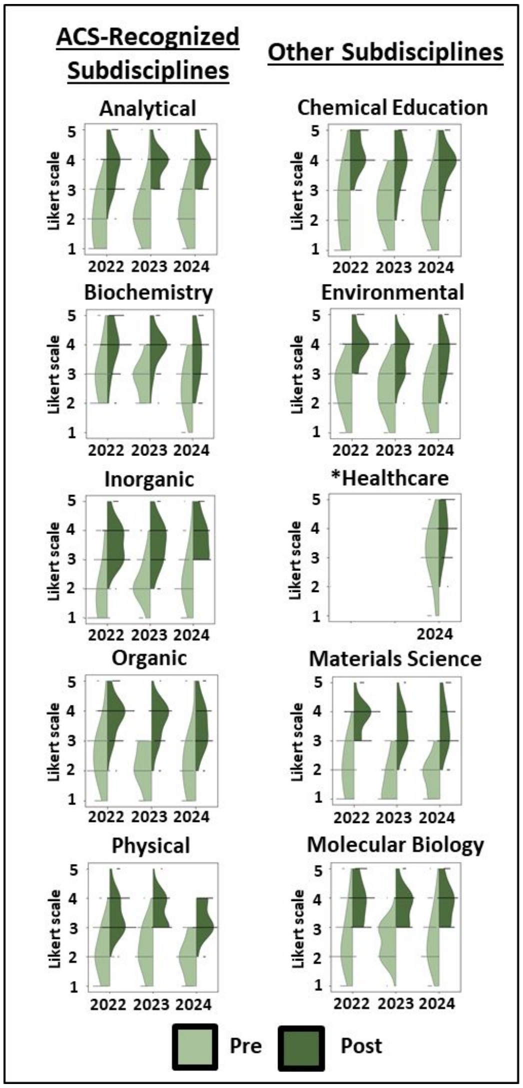

Figure S5: Year-to-year comparisons of survey responses to the Understanding survey items. Pre-seminar responses are shown in light green, post-seminar responses are shown in dark green.

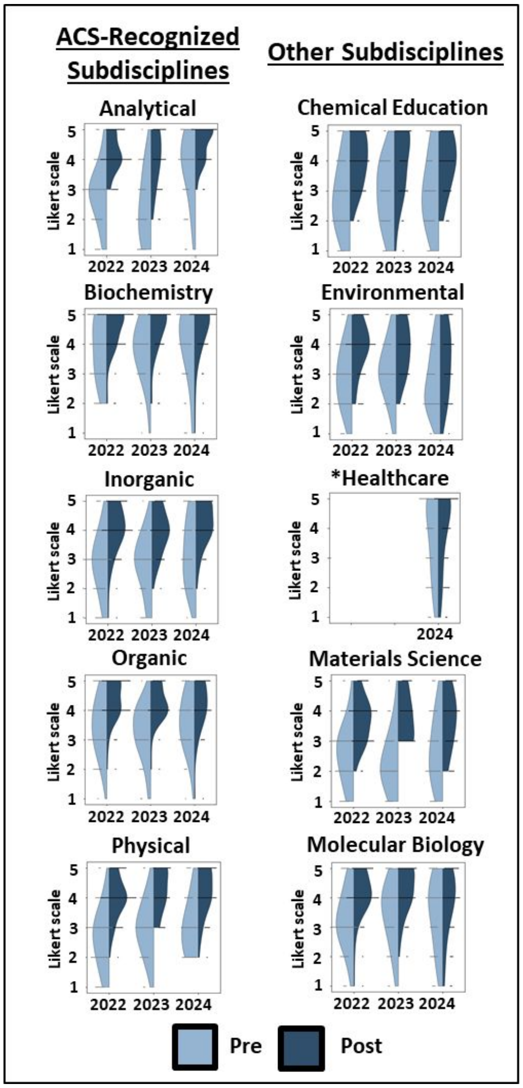

Figure S6: Year-to-year comparisons of survey responses to the Importance survey items. Pre-seminar responses are shown in light blue, post-seminar responses are shown in dark blue.

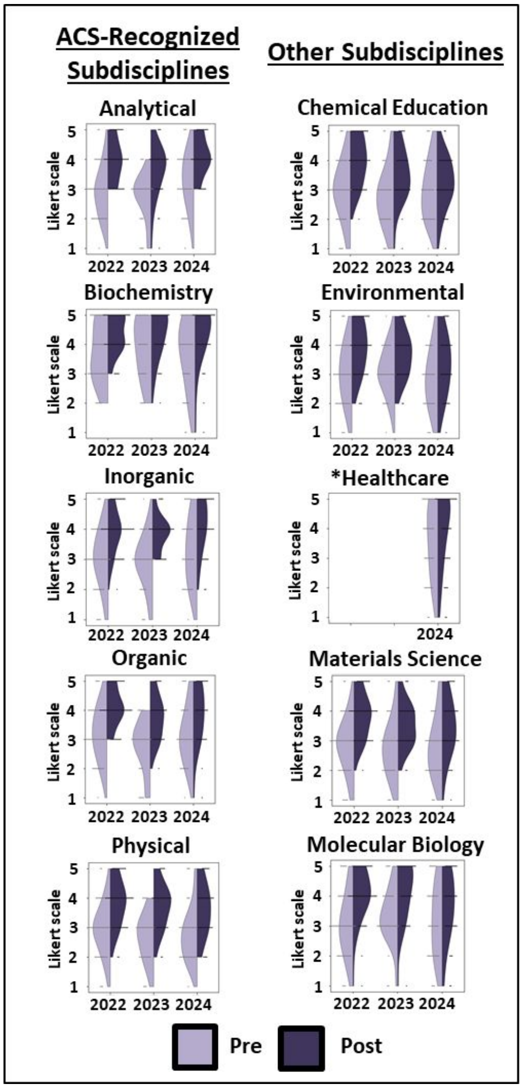

Figure S7: Year-to-year comparisons of survey responses to the Satisfaction survey items. Pre-seminar responses are shown in light purple, post-seminar responses are shown in dark purple.
